# Morphology of the Amazonian Teleost Genus *Arapaima* Using Advanced 3D Imaging

**DOI:** 10.3389/fphys.2020.00260

**Published:** 2020-04-27

**Authors:** Miriam Scadeng, Christina McKenzie, Weston He, Hauke Bartsch, David J. Dubowitz, Dominik Stec, Judy St. Leger

**Affiliations:** ^1^Department of Radiology, University of California, San Diego, San Diego, CA, United States; ^2^Department of Anatomy and Medical Imaging, University of Auckland, Auckland, New Zealand; ^3^Department of Pathobiology, University of Guelph, Guelph, ON, Canada; ^4^NOVA Southeastern University, Fort Lauderdale, FL, United States; ^5^Mohn Medical Imaging and Visualization Centre, Haukeland University Hospital, Bergen, Norway; ^6^Department of Biomedical Sciences, College of Veterinary Medicine, Cornell University, Ithaca, NY, United States

**Keywords:** air-breathing, arapaima, morphology, imaging, pirarucu, osteoglossid, MRI

## Abstract

The arapaima is the largest of the extant air-breathing freshwater fishes. Their respiratory gas bladder is arguably the most striking of all the adaptations to living in the hypoxic waters of the Amazon basin, in which dissolved oxygen can reach 0 ppm (0 mg/l) at night. As obligatory air-breathers, arapaima have undergone extensive anatomical and physiological adaptations in almost every organ system. These changes were evaluated using magnetic resonance and computed tomography imaging, gross necropsy, and histology to create a comprehensive morphological assessment of this unique fish. Segmentation of advanced imaging data allowed for creation of anatomically accurate and quantitative 3D models of organs and their spatial relationships. The deflated gas bladder [1.96% body volume (BV)] runs the length of the coelomic cavity, and encompasses the kidneys (0.35% BV). It is compartmentalized by a highly vascularized webbing comprising of ediculae and inter-edicular septa lined with epithelium acting as a gas exchange surface analogous to a lung. Gills have reduced surface area, with severe blunting and broadening of the lamellae. The kidneys are not divided into separate regions, and have hematopoietic and excretory tissue interspersed throughout. The heart (0.21% BV) is encased in a thick layer of lipid rich tissue. Arapaima have an unusually large telencephalon (28.3% brain volume) for teleosts. The characteristics that allow arapaima to perfectly exploit their native environment also make them easy targets for overfishing. In addition, their habitat is at high risk from climate change and anthropogenic activities which are likely to result is fewer specimens living in the wild, or achieving their growth potential of up to 4.5 m in length.

## Introduction

**FIGURE 1:**
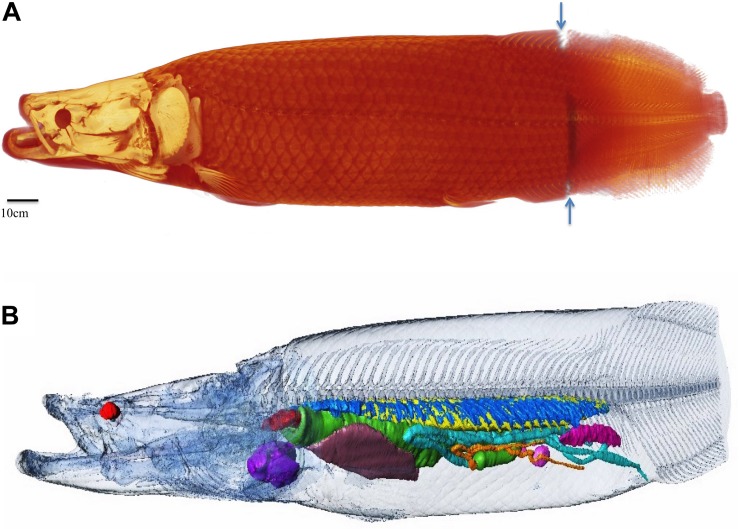
Overview of specimen one. In all figures the traditional radiological convention is adopted with rostral end of fish to the left of the page for sagittal and coronal images and 3D reconstructions. For axial images, left side of image is right side of fish. Right (R), left (L), dorsal (D), ventral (V) marked for clarity. **(A)** 190 cm long female arapaima weighing 70.5 kg, volume of 68.85lt. The average density of the whole specimen is 1.02 g/cm^3^. Whole fish surface reconstructed from CT and MRI data (CT cranial 85% MRI caudal 15% as demarcated by blue arrows). **(B)** Semi transparent surface outline showing relative position of internal organs and skeleton reconstructed from CT data. Heart: purple, liver: brown, spleens: pink, ovary: magenta, esophagus and stomach: green, gut: turquoise, pyloric caeca: orange, respiratory tissue: blue, air in gas bladder: yellow, esophageal sphincter: red. Axial skeleton head and body surface: semi-transparent.

The arapaima, or pirarucu, is an air-breathing osteoglossid teleost native to the Amazonian floodplains, which can reach sizes of up to 4.5 m in length and 200 kg in weight (Graham, 1997; Castello, 2008) (Figure 1). As a genus, *Arapaima* are morphologically, biologically, taxonomically and commercially important.

Arapaima exhibit bimodal breathing, using both gills and a respiratory swim bladder to exchange gases with its environment (Gonzalez et al., 2010; Fernandes et al., 2012). If denied access to air, adult arapaima will drown within 10 min, making them obligate air-breathers (Farrell and Randall, 1978). Until they are approximately 9 days old (18 mm), arapaima exclusively breathe water (Graham, 1997). As they mature, arapaima undergo extensive anatomical and physiological changes, including atrophy of the gills, to become air-breathers (Brauner et al., 2004).

Sometimes referred to incorrectly as “lungs,” the highly specialized respiratory gas bladder of the adult arapaima runs along the dorsal surface of the coelomic cavity (Brauner et al., 2004; Daniels et al., 2004; Fernandes et al., 2012). Many fish possess gas bladders, which can be used for a variety of purposes including buoyancy, sound production and respiration. Many of the more derived teleosts, such as the rainbow trout (*Oncorhynchus mykiss*), a common fish model, have gas bladders that are a smooth bag-like structure (Hall, 1924). Even bichirs (Cladistia), which use a gas bladder for respiration like an arapaima, have a simple gas bladder (Perry et al., 2001). In arapaima, the respiratory gas bladder is complex and subdivided into many irregular ediculae to hugely increase surface area. The lining epithelium is similar to that seen in the gills of non-air-breathing fish (Fernandes et al., 2012). Similar to the majority of the actinopterygii, arapaima use a four-stroke buccal pump to move gas in and out of the respiratory gas bladder, rising to the surface to take a breath approximately every 4 min (Farrell and Randall, 1978; Brainerd, 1994). The respiratory gas bladder is not a true lung, as seen in sarcopterygians such as lungfish (Dipnoi) and tetrapods, because it is dorsal, unpaired and has a different ontological origin (Perry et al., 2001).

Taxonomic classification of arapaima species is a contentious issue. Until recently, it was believed *Arapaima gigas* was the only species (Stewart, 2013b). There are now five recognized species: *A. mapae, A. gigas, A. leptosoma, A. agasizii*, and *A. arapaima*, with the last being discovered as recently as 2013 (Stewart, 2013a).

Although all arapaima were thought to be *A. gigas*, that species is now only known from the museum specimen holotype in Paris (MNHN a-8837). The Amazon is a hotbed of biodiversity due to the variety of microenvironments, allowing for evolution in isolation, and there are likely still more arapaima species to be discovered (Castello et al., 2011; Stewart, 2013a). Aside from presenting an interesting taxonomical conundrum, this poses an important conservation issue as only *A. gigas* is protected under the International Union for Conservation (ICUN) Red List and the Convention on International Trade of Endangered Species (CITES) Appendix 2 (Castello and Stewart, 2010).

Advanced imaging, including computed tomography (CT) and magnetic resonance imaging (MRI), is becoming an increasingly popular means of collecting detailed morphological data from threatened and difficult to acquire species, that can be easily disseminated amongst scientists (Corfield et al., 2008; Berquist et al., 2012; Ponganis et al., 2015; Wright et al., 2016, 2017; York et al., 2018). These techniques allow for excellent imaging of tissue as it exists *in situ*, without disruption from autopsy. By combining the results of advanced imaging and 3D modeling, with detailed examinations using histologic tissue review, we can evaluate the whole animal from a cellular to a gross morphological scale.

## Materials and Methods

### Specimens

A 190cm long female arapaima weighing 70.5 kg was found dead in an aquarium enclosure. The primary findings on necropsy were fibrosing cardiomyopathy, 80 ml of serous pericardial fluid, and 400 ml of sero-hemorrhagic abdominal fluid. The effusions were likely related to the cardiac condition, the cause of which is unknown. This animal, the primary specimen or Specimen One, was examined using multiple modalities for anatomical investigation.

To enhance the histological investigation, three additional arapaima were examined for more detailed histologic information. Specimen Two was an 80 cm long juvenile male, which died in 2008 under anaesthesia. It was used for gill, air bladder and heart histology. Specimen Three was a 140 cm long sub-adult male that jumped from an enclosure in 2007, and was used for kidney histology. Specimen Four was a female arapaima, which died under anaesthesia while under-going enucleation, and was used for brain and digestive system histology.

### Species Identification

Given the many uncertainties surrounding arapaima taxonomy we do not make definitive species identifications for any of our specimens, therefore our findings are representative of the genus *Arapaima*. The morphological indicators that differentiate between species include measurements such as tooth numbers, fin ray numbers and orbit diameter, vary based on body size; there is little interspecies difference in internal anatomy, which is the focus of this paper (Stewart, 2013b).

### Data Collection and Quantitative Analysis

Imaging was performed on the Specimen One within 24 h of death. Necropsy was performed the following day at the facilities of SeaWorld San Diego. Histological slides were prepared using hematoxylin and eosin stain by San Diego Pathologists Medical Group, Inc.

T1 and T2 MR data was collected of the entire fish using a 3 Tesla MRI scanner (GE Sigma HDx MRI scanner – GE Medical Systems, Milwaukee, WI, United States) at the University of California, San Diego Center for Functional MRI. Due to the size of the specimen all imaging was acquired using the machine body coil, and in several sections, limited by the useable length of the imaging coil. T2-weighted images were acquired using a 2D fast spin echo imaging sequence with the following protocol parameters: echo time = 116 ms; repetition time = 3500 ms; averages; in-plane matrix = 512 × 512; in plane resolution = 0.55 × 0.55 mm; slice thickness = 4 mm. T1-weighted images were acquired using a 3D fast spoiled gradient echo sequence with the following protocol parameters: echo time = 4.3 ms; repetition time = 10.2 ms; averages; inversion time 450 ms, in-plane matrix = 512 × 512, in plane resolution = 0.43 × 0.43 mm; slice thickness = 1 mm. Distortion correction is performed automatically using GE software, and the length of the acquisition was limited to the linear portion of the gradient coil, with care taken to overlap the acquired segments. This ensured the whole length of the fish was acquired while avoiding either missing segments or distorted data.

CT data was collected using a GE lightspeed Discovery 750HD CT scanner (GE Medical Systems, Milwaukee, WI, United States). Scanning parameters were 120 kV, 226MA. In plane resolution 0.98 × 0.97 mm; slice thickness 1.25 mm. As the length of the specimen was longer than the CT machine table, the tip of the caudal fin was not imaged, though the surface model of the whole fish was reconstructed using MRI data of this region for accurate total body volumes (Figure 1).

Segmentation was manually performed using AMIRA software (FEI Visualization Sciences Group, Burlington, MA, United States). Segmentation was based on image grayscale intensity and *a priori* knowledge of anatomical characteristics of ostoglossid fish, spatial relationships and external landmarks. 3D models of the anatomy were constructed from the segmented imaging data.

Morphometric measurements were taken as follows: heart and vessel wall and lumen measurements were taken from MR data at three representative points and averaged. Tooth counts are an approximation. They were obtained from CT images, and as the teeth are similar in size to the image resolution this made some individual teeth difficult to define separate from neighboring teeth. The tooth counts for the dentary include only a single ramus. Orbit diameter was measured from CT images at the widest point. Interorbital width was measured from CT images from the most medial points of each orbit. Caudal peduncle length was measured from necropsy photos as the distance between the caudal insertion of the anal fin and the cranial insertion of the caudal fin. Anal fin rays were counted using a combination of CT and MR images. Total density of Specimen 1 was calculated using total body weight at necropsy divided by the total body volume measured using segmented imaging data.

Hematoxylin and eosin staining is done per Stevens (1982): tissues are fixed, dehydrated and embedded in melted paraffin wax, which facilitates microtoming into thin slices. After rehydrating, hematoxylin and a metallic salt is applied to the tissue, which is then rinsed in a weak acid solution, followed by rinsing in mildly alkaline water. This stains cell nuclei blue. The tissue is then stained with eosin, which stains the extracellular matrix and cytoplasm pink.

## Results

### Gill Morphology

**FIGURE 2:**
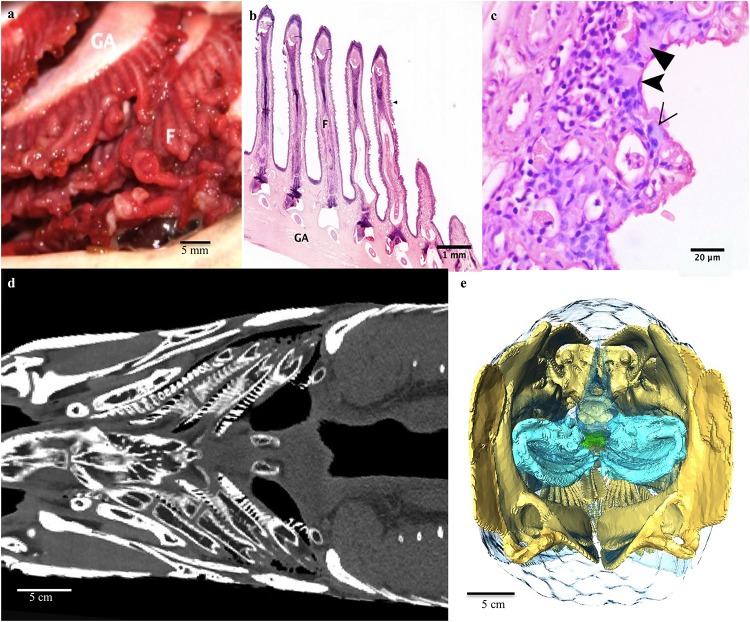
Respiratory system: The gill arches and filaments. **(a)** On each side there are four gill arches (GA) with associated cartilaginous filaments (F). **(b)** Photomicrograph of the boney gill arch, cartilaginous filaments. Note extensive blunting and broadening of the lamellae. **(c)** A higher magnification light micrograph of the lamellae of a gill filament. The epithelium of the lamellae is made up of ionocytes (filled arrowheads), mucocytes (empty arrowhead) and pavement cells (notched arrowhead). Note the collapse of the pillar cell system. **(d)** Coronal CT slice at level of the base of mouth demonstrating the gill arches and filaments. **(e)** 3D reconstruction of gill arches (blue) from CT as seen from caudal end of fish looking cranially into the fish.

There are four pairs of gill arches with accompanying filaments (Figure 2d). The filaments are short, squat and have only rudimentary lamellae. The lamellae are extremely blunt and broad compared to those of the gills of water-breathing fish and the pillar system is absent (Figure 2b). There is a slight curl to the distal portions of the filaments on necropsy, but this appears to be artifact as it not present on histology and may have been due to post mortem necrosis (Figure 2a). The lamellar epithelium is comprised of mucocytes, ionocytes, and pavement cells (Figure 2c). The gills subjectively appear to be less highly vascularized than most water-breathing fish.

### Esophageal Sphincter and Respiratory Gas Bladder

**FIGURE 3:**
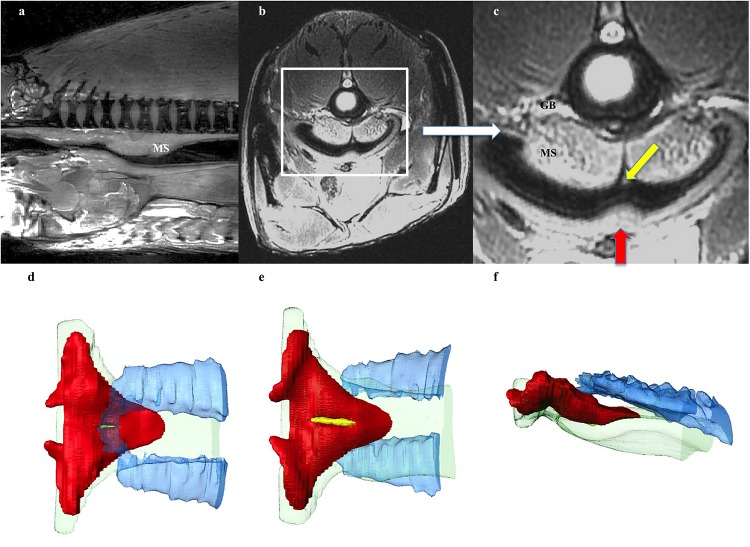
Respiratory system: Esophageal Sphincter. **(a)** Midline sagittal, and **(b,c)** axial MRI image at level of upper esophagus through the muscular sphincter (MS), positioned in the dorsal aspect of the esophagus. White box on **(b)** indicates enlarged section **(c)**. The sphincter is a triangular, disc-like shaped muscle through which air moves between the esophagus and the rostral aspect of the gas bladder (GB), which is not inflated at this level. A tiny longitudinal strip of tissue seen overlying the opening (yellow arrow in **c**), may act as a valve regulating the flow of air, or preventing water/food from entering the GB. In addition a localized thickening of tissue (red arrow) sited on the opposing side of the esophagus, possibly occludes the slit opening when the surfaces of the esophagus are opposed. **(d,e)** 3D reconstruction of muscular sphincter from CT data demonstrating relationship between the esophagus and the GB. Red: muscular sphincter, blue: rostral end of GB, green: esophagus, yellow: strip of tissue in valve opening. **(d)** From above sphincter, **(e)** from below sphincter, **(f)** lateral view.

**FIGURE 4:**
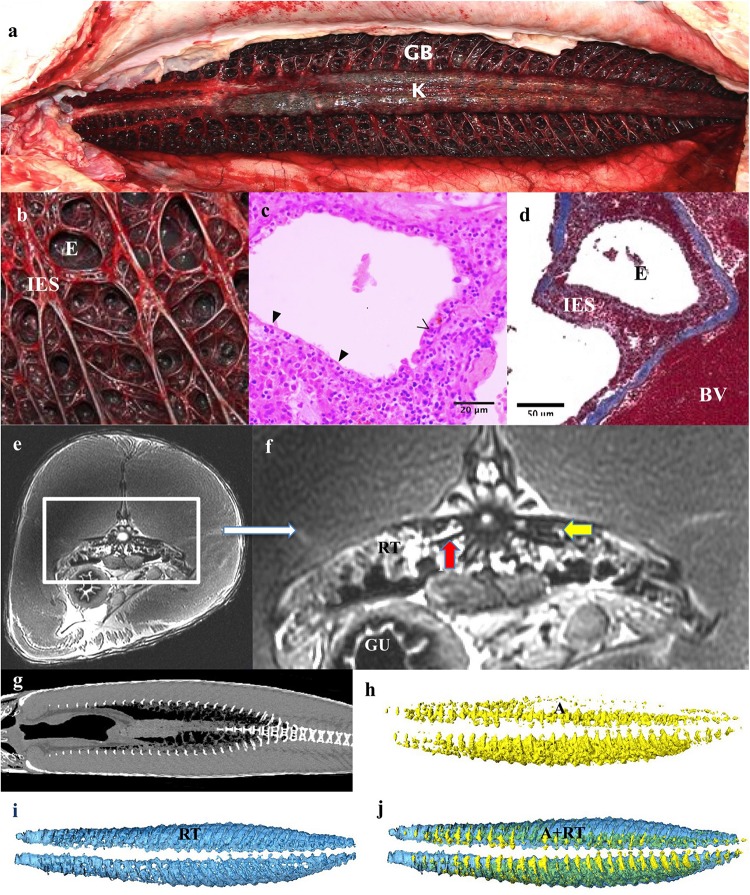
Respiratory system: Respiratory gas bladder. **(a)** The gas bladder (GB) and respiratory tissue (RT) runs the length of the dorsal coelomic cavity and encompasses the kidneys (K). **(b)** The dorsal surface is covered by a mat of highly vascular tissue comprising of ediculae and inter-edicular septa. The inter-edicular septa are created by trabeculae made of smooth muscle and connective tissue. The interior surfaces of the gas bladder that are in contact with air are all covered with respiratory epithelium consisting of pavement and columnar epithelial cells. **(c,d)** A light micrograph stained with Masson’s Trichrome showing a blood vessel (BV), inter-edicular septa (IES) and ediculae (E). Collagen is stained blue. **(e,f)** Axial and zoomed axial MRI of respiratory tissue at level of mid of the GB demonstrating respiratory tissue (RT), gas in air bladder (A), Gas in stomach (G). The kidneys (K) are seen suspended in the GB, vertebrae (V) and gut (GU). Vertebral bodies appear to have spicules emanating from them (red arrow) – Similar appearance seen on CT in Figure 10. This feature is presumably to increase surface area for gas exchange and have not previously been described in this species. **(g)** Coronal CT scan at level of GB demonstrating relationships of contents of GB. **(h–j)** 3D reconstructions from CT data. **(h)** Air (A) fraction in GB (yellow), **(i)** Vascular respiratory tissue fraction (blue). **(j)** Reconstructions of GB showing the relationship of A to RT.

**FIGURE 5:**
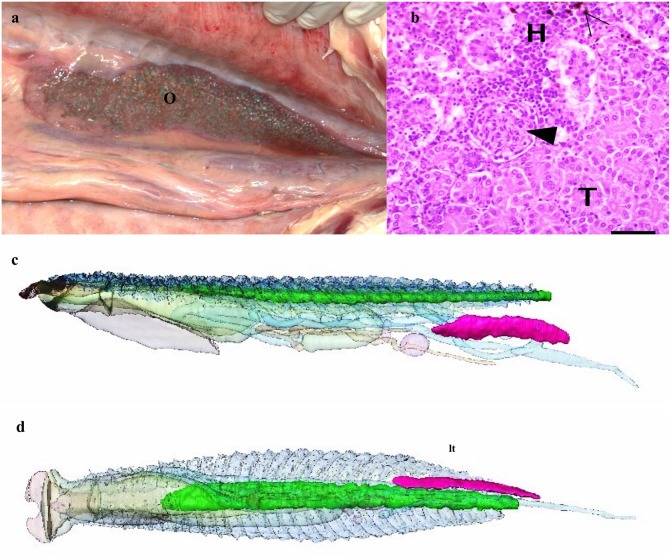
Ovary and kidneys. **(a)** Gross anatomy of ovary (O) in the peritoneal cavity. **(b)** Kidney- light micrograph showing hematopoietic tissue (H), renal tubules (T), capillary tuft (arrowhead) and melanomacrophages (empty arrowhead). **(c)** 3D reconstruction from CT data of kidneys (green) and ovary (magenta) as seen from left side and **(d)** from below looking up. Other organs as described above but semitransparent for orientation.

The respiratory gas bladder is unpaired and runs the dorsal length and width of the coelomic cavity, encompassing the kidneys (Figures 3, 4, 5). The gas bladder is connected to the esophagus cranially via a substantial dorsal muscular sphincter (Figures 3a–f). The sphincter is largely a triangular, disc-like shaped muscle measuring 5cm in length. In the midline is a longitudinal slit-like opening that measures 24 mm in length. It is through this opening that air moves between the esophagus and the rostral aspect of the gas bladder (GB), which was not inflated at this level. A tiny longitudinal strip of possible tissue is seen on CT and MR imaging overlying the opening (yellow arrow in 3c), and this may act as a valve regulating the flow of air, or preventing water/food from entering the GB. In addition a localized thickening of tissue (red arrow Figure 3c) sited on the opposing side of the esophagus, possibly occludes the slit opening when the surfaces of the esophagus are opposed.

The dorsal aspect of the gas bladder is highly vascularized and appears dark red on necropsy (Figures 4a,b). Grossly, the surface of the gas bladder appear as a disorganized, dense web of tissue with air compartments, or ediculae, of varying size. The inter edicular septa are comprised of muscular supportive structures and blood vessels (trabeculae) with a layer of respiratory epithelium covering areas in contact with gas (Figures 4b–d). This epithelium is comprised of pavement cells and columnar cells. The respiratory tissue measured 60 mm at its thickest part. The total volume of the respiratory tissue is 785 ml excluding the air component.

At the time of CT and MR data collection, the gas bladder was almost completely collapsed, with much of the air residing within the mass of respiratory tissues. On the CT data of Specimen One the right side of the gas bladder was more inflated than the left side (total gas volume 564 ml, Table 1). The asymmetry in air volume is likely due to the left side being dependant during transportation of the specimen. The T2 weighted MR image (water has high signal intensity on T2-; Figures 4e,f) demonstrates that there is some fluid within the gas bladder, which highlights the visualization of the inter-edicular septa. The respiratory gas bladder makes up 1.96% of the total volume of this arapaima (Table 1).

**TABLE 1:** Body structure volumes in milliliters (ml) and as percent of whole fish.

**Structure**	**Volume (ml)**	**% of total**
Muscle/other soft tissue	56,860.00	82.57%
Head/axial skeleton	3,757.00	5.46%
Scales	4,214.00	6.12%
Air in gas bladder	564.40	0.82%
Respiratory tissue	784.70	1.14%
Kidney	243.50	0.35%
Esophageal sphincter	100.20	0.15%
Esophagus and stomach	1,361.00	1.98%
Gut	267.70	0.39%
Blind caeca 1	15.42	0.02%
Blind caeca 2	19.32	0.03%
Liver	392.70	0.57%
Spleens	24.99	0.04%
Heart	144.30	0.21%
Pericardial fat	42.52	0.06%
Ovary	55.62	0.08%
Brain	6.74	0.01%
Eye1	6.05	0.01%
Eye 2	5.97	0.01%
Lenses	0.73	0.001%

*Structures segmented from CT and MRI data. Numbers corrected to 4 significant figures.*

### Kidneys and Ovary

The kidneys are paired and run medially along the spine within the respiratory gas bladder (Figures 4e–g, 5c,d) and occupy approximately the caudal three quarters of this space with the respiratory tissue on each side. The kidneys are in contact with each other along their length. They comprise 0.35% of the total volume of the arapaima (Table 1). There is no differentiation into a hematopoietic “head” and excretory “trunk,” as hematopoietic cells and nephrons are interspersed throughout the parenchyma. The nephron consists of a renal corpuscle and renal tubule, emptying into a collecting duct.

Specimen One is an adult female with an inactive left ovary and no right ovary. The ovary is long, thin, and flat and tapers to a point cranially. The left ovary comprises 0.08% of the total volume of the arapaima (Figures 5a,c,d).

### Heart and Vasculature

**FIGURE 6:**
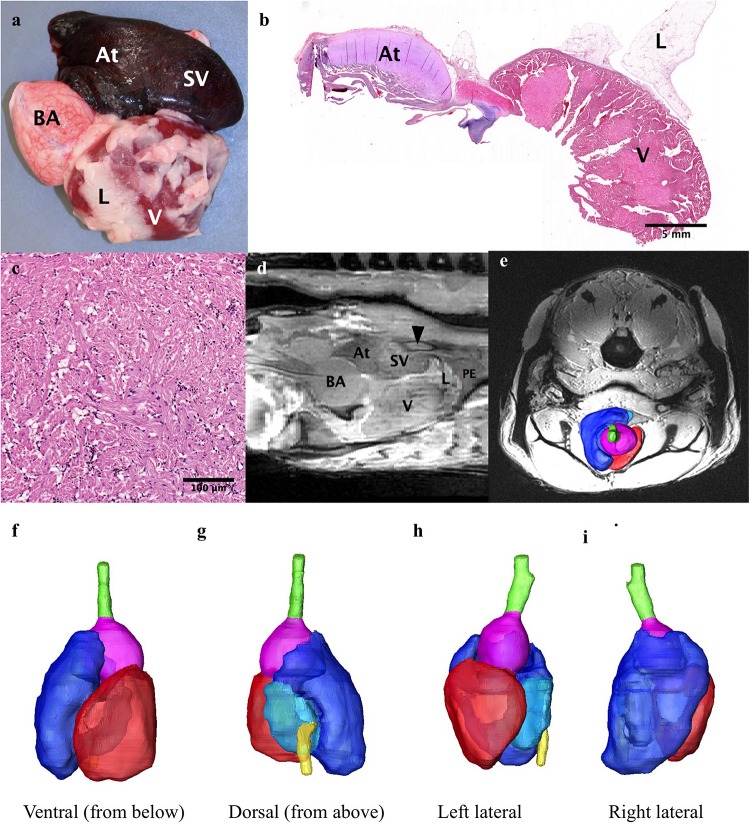
The heart. **(a,b,d)** The heart consists of the sinus venosus (SV), the atrium (At), the ventricle (V) and the bulbus arteriosis (BA). Some of the substantial lipid rich connective tissue that surrounds the heart can be seen (L). **(c)** Photomicrograph of the ventricular myocardium. **(d)** Sagittal MRI of the heart showing the ductus of Cuvier (arrowhead) connecting to the sinus venosus. A small amount of pericardial effusion (PE) can be seen at the apex of the heart. **(e–i) e-**3D reconstruction of heart superimposed on axial MRI for orientation of heart. **(f)** Ventral (seen from below), **(g)** Dorsal (from above), **(h)** Left lateral, **(i)** Right Lateral. Hepatic vein: yellow, sinus venosus: light blue), atrium: dark blue, ventricle: red, bulbus arteriosus: purple.

The heart is found encased in a thick layer of lipid rich connective tissue just caudal to the gill arches. The heart consists of the sinus venosus, atrium, muscular ventricle, conus arteriosus, and bulbus arteriosus (Figures 6a–f). Blood enters the heart via the ductus Cuvier, which is fed by the hepatic and renal portal systems. Blood exits the heart to the dorsal aorta toward the gills. Only the heart parenchyma was included in segmentation; the lumen was excluded. The heart musculature constitutes 0.21% of the total volume of the arapaima, with the majority of the heart volume being the ventricle (Table 2). The ductus Cuvier has a diameter of 6.3 mm where it meets the sinus venosus. The wall of the sinus venosus is 1.1 mm thick and the atrium is 2.0 mm thick. The thickness of the ventricular wall varies between 7.8 and 14.8 mm, depending on the musculature. The lumen of the ventricle is very narrow in MR images, indicating a contracted state at the time of death. The conus arteriosus is a distinct entity between the ventricle and the bulbus arteriosus and supports the conus valve (Figure 5a). The bulbus arteriosus has a thick fibroelastic wall, measuring 1.9 mm thick, with tissue folds protruding into the lumen, which is 19.9 mm at the thickest point. Specimen one has moderate pericardial effusion, which can be seen in the MR images (Figure 6d).

**TABLE 2:** Cardiac structure volumes in milliliters (ml) and as percent of whole heart volume.

**Structure**	**Volume (mls)**	**% of total**
Ventricle	37.28	30.1%
Bulbus arteriosus	16.79	13.6%
Atrium	58.51	47.2%
Sinus venosus	11.28	9.1%

*Structures segmented from MRI data. Numbers corrected to 4 significant figures.*

### Gastrointestinal System

**FIGURE 7:**
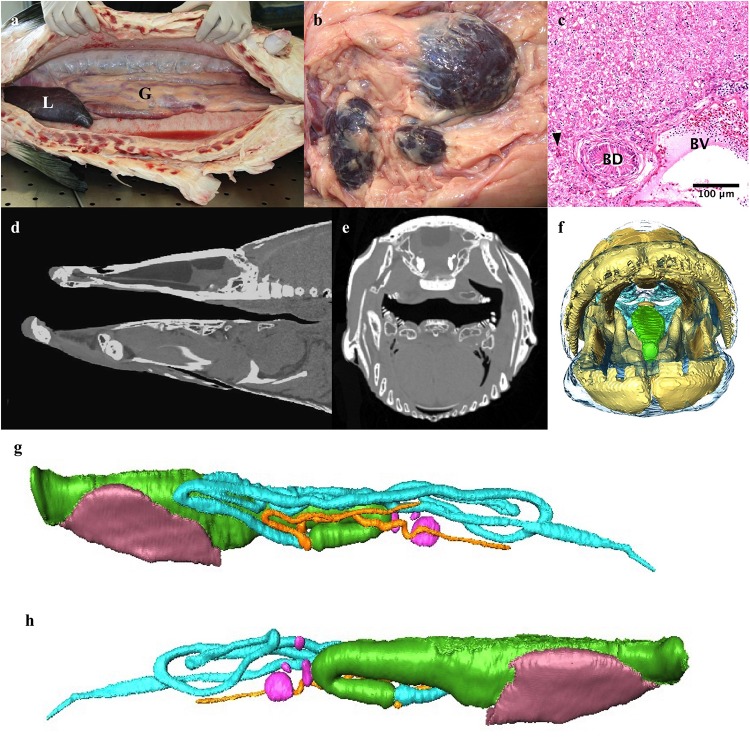
The digestive system. **(a)** Abdominal cavity with liver (L) and gut (G). **(b)** Several spleens. **(c)** Light micrograph of liver showing a bile duct (BD), blood vessel (BV), sinusoids (arrowhead) and hepatocytes (top half of image). **(d,e)** Sagittal midline and axial CT slice through head demonstrating how effective crushing of prey occurs between boney tongue and roof of mouth. **(f)** 3D reconstruction of head looking into mouth highlighting boney tongue (green). **(g,h)** 3D model of gut and liver reconstructed from CT data. The gut of the osteoglossomorpha is distinct from that of other fishes in that the intestine passes posteriorly to the left of the esophagus and stomach (Nelson, 1972) rather than to the right. **(g)** left lateral, **(h)** Right lateral. Esophagus and stomach: green, intestinal loops: turquoise, blind ending pyloric caeca originating from proximal gut: orange, liver: brown, spleens pink.

The teeth of the dentary, premaxillary and maxillary bones are simple, conical, approximately 2 mm long and present in single rows. There are 36 dentary teeth and 34 maxillary teeth. The basibranchial tooth plate, or boney tongue, is covered in fine villiform lingual teeth (Figures 7d–f).

The esophagus is approximately 7 cm long and has a large dorsal muscular sphincter connecting it to the respiratory gas bladder as described above (Figure 3). The esophagus connects to a muscular stomach. The stomach has a thin tunica serosa, a thick tunica muscularis with perpendicular and circular muscle fibers, a tunica submucosa and a tunica mucosa with both gastric glands and goblet cells. There are two blind-ended pyloric ceca. The intestine passes posteriorly to the left of the esophagus and stomach rather than to the right (Figures 7g,h). The intestines loop back and forth four to five times within the coelom before terminating at the rectum. The gastrointestinal tract – esophagus –rectum, constitutes 2.56% of the total body volume (excluding gas volume).

The liver has one trough shaped lobe that lies ventral to the gastrointestinal tract in the cranial aspect of the coelomic cavity and comprises 0.57% of the total body volume (Figure 7a and Table 1). It is dark reddish-brown in color. The hepatocytes are hexagonal in shape, vacuolated, and have a round centrally located nucleus with a darkly staining nucleolus (Figure 7c). A network of sinusoids runs between the hepatocytes. There are many larger blood vessels within the liver parenchyma. The bile ducts are lined with a single layer of columnar epithelial cells. There are several spleens (Figure 7b).

### Brain

**FIGURE 8:**
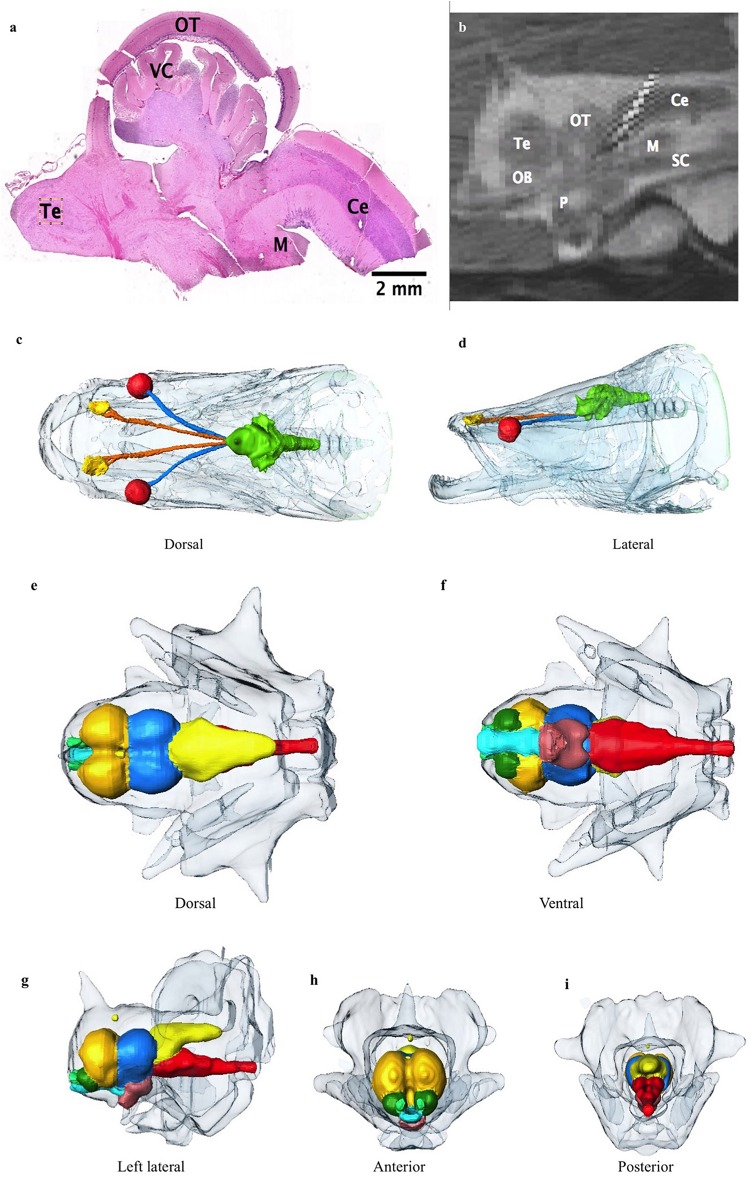
**(a)** Photomicrograph showing the cerebellum (Ce), medulla (M), optic tectum (OT), valvula cerebelli (VC) and telencephalon (Te). **(b)** Sagittal midline T2 MR image through brain showing the cerebellum, optic tectum, telencephalon, olfactory bulb (OB), pituitary (P), spinal cord (SC) and medulla. Surrounding high signal is expansive cerebro-spinal fluid (CSF) space in which the brain is suspended. **(c,d)** Dorsal and lateral view 3D reconstructions of the head from CT data showing the extent of the CSF space encasing the brain (green), optic nerves (blue) and olfactory nerves (orange). Eyes are red, and lining of olfactory pits yellow. Other bony head structures are semi transparent head. **(e–i)** 3D reconstructions of the brain with large and complex CSF space surrounding brain (semitransparent surface). **(e)** Dorsal, **(f)** Ventral, **(g)** lateral, **(h)** anterior, and **(i)** Posterior view. Olfactory bulbs: green, telencephalon: dark yellow, hypothalamus: brown, optic tectum: blue, cerebellum: light yellow, medulla and proximal spinal cord: red, and proximal optic tract: turquoise.

The rostral-most structures are the paired olfactory bulbs, which are attached caudally to the cerebral hemispheres (Figures 8a–i). The telencephalon is large and well developed compared to the majority of fish, especially other osteoglossids; it has well differentiated right and left lobes. The diencephalon is primarily composed of the preoptic area. The mesencephalon is predominately optic tectum. Caudal to this is the cerebellum and the medulla. The brain makes up only 0.01% of the total body volume (Table 3) and the largest subdivision is the telencephalon at 28.3% of brain volume. The brain case is significantly larger than the brain itself (Figures 8b–i). There is a large and complex cerebro-spinal fluid (CSF) space around the brain which measures more than 3 times (323%) the volume of the brain itself (Figure 8b), and the space around and anterior to the CSF space is filled with fatty connective tissue.

**TABLE 3:** Brain structure volumes in milliliters (ml) and as percent of whole brain volume.

**Structure**	**Volume (ml)**	**% of total**
CSF	21.59	323%
Brainstem	0.86	12.88%
Cerebellum	1.09	16.37%
Optic tectum	1.86	27.89%
Diencephalon	0.38	5.65%
Telencephalon	1.89	28.31%
Olfactory bulb	0.25	3.76%
Optic tract	0.17	2.59%
Pineal	0.01	0.15%
Olfactory tract	0.04	0.64%
Pituitary	0.12	1.77%

*Cerebrospinal fluid (CSF) volume outside the brain also included. Structures segmented from MRI data. Numbers corrected to 4 significant figures.*

### Musculoskeletal and Integument

**FIGURE 9:**
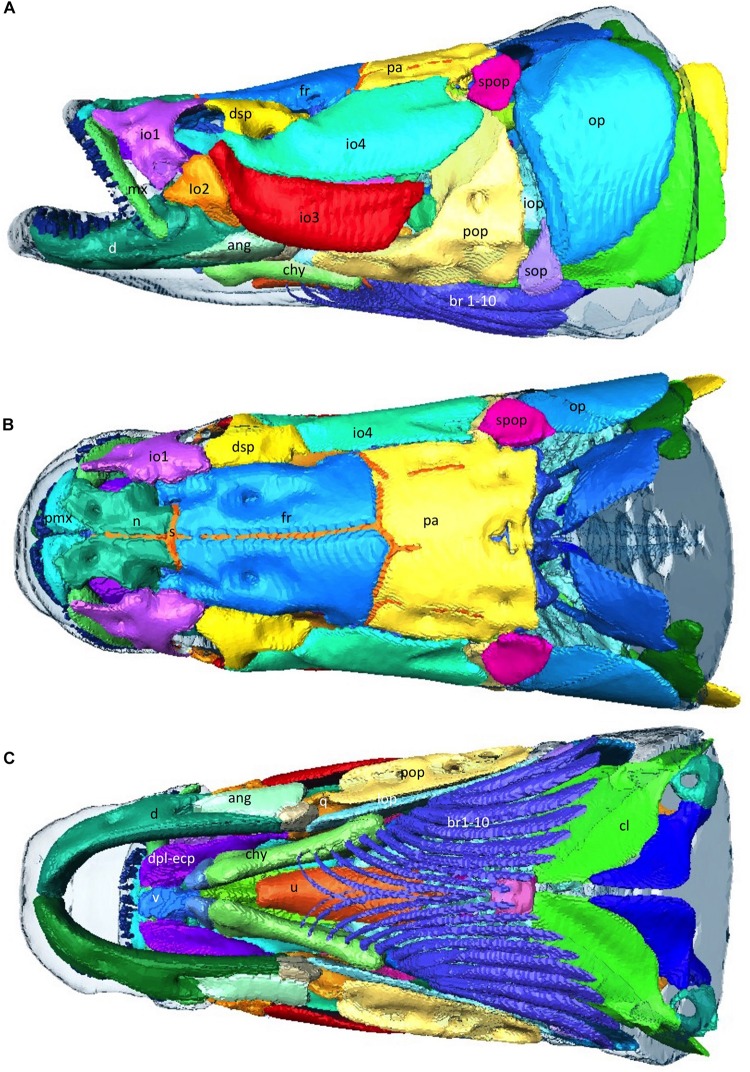
Musculoskeletal -Head. **(A–C)** 3D reconstructions from CT data, of the external bony armor plates of the head with integument overlain as transparent surface. Abbtp, anterior basibranchial toothplate; ang, angular; ant, antorbital; br, branchiostegal rays; chy, Ceratohyal; cl; d, dentary; dsp, dermosphenotic; fr, frontal; io 1–4, infraorbital 1–4; iop, interopercular; iop, infraopercular; mx, maxilla; n, nasal; op, opercle; pa, parietal; pmx, premaxilla; pop, preopercle; q, quadrate; s, suture; sop, subopercle; spop, suprapreopercle; u, urohyal; v, vomer. (Bones referenced as per Steward 2013).

The cranium is heavily ossified and well protected consisting of 2 main layers of overlapping bones. (Figures 9A–C) The mouth opens upwards toward the surface. There are indentations in the cranium around pores to the acousticolateralis system (Figure 9A). The orbit diameter is 1.83% of total length and the interorbital width is 6.3% of total length.

**FIGURE 10:**
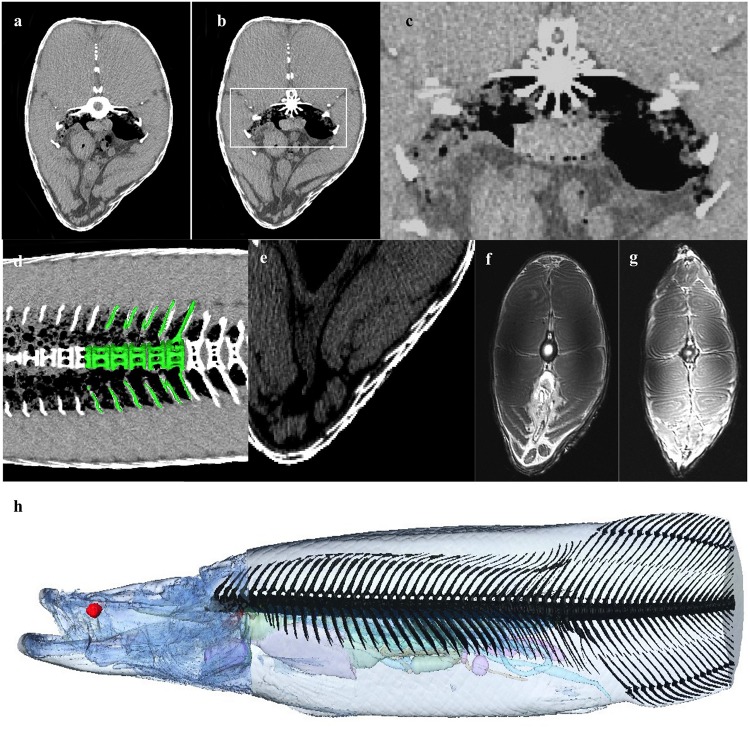
Musculoskeletal and Integument. **(a)** Axial CT at level of GB demonstrating air partially encased between the bony cortices of the transvers processes of the vertebral bodies, and **(b,c)** shows spiculation of the vertebral body, possibly to increase surface area of respiratory epithelium exposed to air. **(d)** 3D rendering of vertebrae on background of coronal CT slice showing air density within part of the in the vertebral body between spicules. **(e)** Axial CT section demonstrating flexible dermal armor of elasmoid scales made from overlapping layers of type 1 collagen and a highly mineralized hydroxyapatite outer layer (Yang et al., 2014). Scales overlap with a thickness up to 9.7 mm. Single scale thickness is approximately 3.4 mm. **(f,g)** The muscle is divided in to left and right epaxial and hypaxial muscle by the vertical and horizontal septa. **(h)** Bony axial skeleton reconstructed from CT data (excluding distal tail).

Bone (skull bones and axial skeleton) comprise 5.45% of the total volume, with skull bones constituting more than half of that, and the integument comprises 6.12% of the total volume. The 79 vertebrae are composed of a centrum, a neural spine and transverse processes. The ventral aspect of the vertebral bodies in the region of the gas bladder are spiculated and interdigitate with the respiratory tissue (Figures 10a–d). Many of the transverse processes of the vertebrae in the region of the gas bladder appear to partially encase air. There are 34 pairs of ribs (see Figure 10h). Arapaima have pectoral fins, pelvic fins, an adipose fin, an anal fin and a caudal fin. The adipose and anal fins are the most substantial, squaring off the peduncle before the caudal fin in a lateral view (Figure 1). Thirty-four anal fin rays support the anal fin and 36 support the adipose fin. The caudal fin is small and triangular with 27 boney rays.

The integument covering the head is green dorsally and white ventrally. There are pores on the mandible and ventral cranium that open to the acousticolateralis system. The scales of the body are thick and gray in color and lighter ventrally, creating heavy armor. Where the scales overlap the thickness is up to 9.7 mm. Single scale thickness is approximately 3.4 mm (Figure 10e). On the caudal trunk, some scales have bright red areas posteriorly which form diagonal lines along the sides of the fish. The amount of red pigment increases caudally. The body and tail are heavily muscled with mostly white muscle. Muscle makes up the majority of the fish (muscle and minor organ/soft tissue constitutes 82.6% of total). The muscle is divided in to left and right epaxial and hypaxial muscle by the vertical and horizontal septa (Figures 10f,g). The muscle is segmented into myomeres.

## Discussion

Arapaima are highly specialized for life in the intermittently hypoxic environment of the Amazon, giving them a competitive edge during times of low water levels (Castello et al., 2011) in which dissolved oxygen can reach 0 ppm (0 mg/l) at night, though anoxic environments only occur in deeper waters (Junk et al., 2015). Many Amazonian fish have developed methods of compensating for hypoxia, such as the pharyngeal diverticula of the swamp eel (*Snybranchus marmoratus*) for air breathing, or the expansion of the lip in the pacu (*Piaractus brachypomum*) to exploit the more oxygen rich surface waters (Val et al., 1998). However, none are as striking or as comprehensive as the changes associated with the respiratory gas bladder in arapaima. The development of obligate air-breathing required the compensatory evolution of several additional morphological and physiological adaptations. In addition to changes in the respiratory organs, such as the gas bladder and gills, there are alterations in renal function and the vascular supply. Together, these morphological specializations allow the arapaima to fill a unique ecological niche in the Amazonian basin and make the arapaima a fascinating anatomical study.

### Respiratory System

Although air-breathing has evolved multiple times in teleosts, no fish has freed itself entirely from using gills while remaining in an aquatic habitat, except the obligate air-breather adult arapaima (Graham, 1997). Arapaima are highly specialized to withstand hypoxia, with 50–100% of their oxygen coming from the air, depending on the oxygenation of the water (Stevens and Holeton, 1978) and size or age. They have higher metabolic rates than most fish and increased aerobic capacities, existing in a state of compensated respiratory acidosis with lower blood pH and higher pCO_2_ levels than most fish (Hochachka et al., 1978b; Brauner et al., 2004; Gonzalez et al., 2010). Despite their reliance on air, arapaima ventilate their gills 16–24 times/minute and only stop during inhalation at the surface (Farrell and Randall, 1978; Stevens and Holeton, 1978) and this may be related to acid base balance and CO_2_ excretion, though they may also maintain use of their gills in order to minimize time spent at the surface, where they are more prone to predation (Kramer et al., 1982).

### Mechanics of Breathing

When arapaima ventilate their respiratory swim bladder, they only break the surface of the water for one second (Farrell and Randall, 1978). The four-stroke buccal pump breathing mechanism of the arapaima results in exchange the contents of their gas bladder (Greenwood and Liem, 1984; Liem, 1989). Farrell and Randall (1978) proposed that effective gas bladder ventilation is due to a diaphragm-like septum that stretches between the body flanks such that, when there is an outward movement of the flanks, the septum is pulled downwards creating negative pressure to fill the gas bladder. But, similar to Greenwood and Liem (1984), we found no evidence of such a structure. As the fish rises toward the surface, gas is moved from the bladder into the buccopharyngeal cavity and out through the opercular slit (Greenwood and Liem, 1984; Liem, 1989). When the mouth comes out of the water, the buccopharyngeal cavity is filled with air and the arapaima sinks below the surface while the buccopharyngeal floor rises and air moves to the gas bladder (Greenwood and Liem, 1984; Liem, 1989).

### Gill Morphology

Like most teleosts, arapaima have four pairs of gill arches with two rows of filaments and associated lamellae which are used for gas exchange, ionoregulation, acid-base balance and nitrogenous waste excretion (Figure 2). Unlike most fish, arapaima gills are drastically decreased in surface area, have shorter lamellae, thicker blood-water barriers and a smaller role in homeostatic functions (Hulbert and Moon, 1978; Brauner et al., 2004; Gonzalez et al., 2010; Ramos et al., 2013). As arapaima mature and become increasingly reliant on air-breathing, the intralamellar spaces fill with a proliferation of chloride cells (more recently known as ionocytes), increasing the thickness of the blood-water barrier, and there is atrophy of the pillar system, decreasing blood supply (Brauner et al., 2004; Da Costa et al., 2007; Ramos et al., 2013). These changes are thought to help decrease the loss of oxygen from the blood into hypoxic waters while still allowing for CO_2_ exchange (Graham, 1997; Brauner et al., 2004; Gonzalez et al., 2010). Ionocytes are usually only found on the edge of the filamental epithelium and function in the reuptake of Cl^–^ and Ca^2+^ ions, and their proliferation may indicate that ion and acid-base regulation is a more important role for arapaima gills than respiration (Brauner et al., 2004; Ramos et al., 2013). In addition to ionocytes, the epithelium of the gills also contains mucocytes (Figure 2C; [Bibr B17][Bibr B57]).

### Respiratory Gas Bladder Morphology

As previously described, we found the gas bladder runs the length of the coelomic cavity, encompassing the kidneys. The dorsal surface is covered by a mat of highly vascular tissue comprising of ediculae and inter-edicular septa. The inter-edicular septa are created by trabeculae made of smooth muscle and connective tissue (Figure 4; Hochachka et al., 1978b; Greenwood and Liem, 1984; Fernandes et al., 2012). The interior surfaces of the gas bladder in contact with air are all covered with respiratory epithelium consisting of pavement and columnar epithelial cells (Figure 4c; Fernandes et al., 2012). Electron microscopy shows pavement cells have fewer mitochondria and are closely associated with capillaries on the basal surface, while the apical surface has short microvilli (Fernandes et al., 2012). The columnar cells have more mitochondria and microvilli (Fernandes et al., 2012). Columnar and pavement cells are connected by tight junctions (Fernandes et al., 2012). The blood-air barrier in arapaima is made up of capillary endothelial cells, the basal lamina and the pavement cells and is so thin it is comparable to avian lungs (Fernandes et al., 2012).

Our respiratory gas bladder volume of 2.56% total volume is substantially less than the 7.86% volume reported in Fernandes et al. (2012) and the 10% mass volume reported in Randall and Farrell (1978). This is due to the fact that the gas bladder in Specimen One were almost completely deflated at the time of advanced imaging (Table 1) and perhaps some variation in measuring techniques. Our measured value of 1.39% volume for the respiratory parenchyma (excluding air within it) is similar to the 2.14% volume reported in Fernandes et al. (2012), lending credence to the under-inflation hypothesis. Even this may be an overestimation as the respiratory tissue could have been congested due to the fibrosing cardiomyopathy. In Fernandes et al. (2012), volume was calculated using stereological point and intersection counting methods using a microscope and computer software in a juvenile arapaima. In Randall and Farrell (1978), gas bladder volume was measured as the volume of water required to fill it, which may have involved abnormal stretching of the organ. The gas bladder parenchyma has a surface to volume ratio of 221 cm^–1^, which is 5–33 times greater than the ratio in other air-breathing fish (Fernandes et al., 2012). For instance, the reported volume of the fully inflated gas bladder in a live *O. mykiss* is 2.65% of total volume (Martinez et al., 2014). The swim bladder of *O. mykiss* is a physostomus membranous sac with a rete mirabile and is used mainly for buoyancy rather than respiration (Hall, 1924). Fully inflated, the gas bladder of the arapaima is likely to appropriate a significant amount of space in the coelomic cavity, reflective of its change in function.

While the respiratory gas bladder of arapaima is highly complex, it is considered primitive because it lacks esophageal specialization and maintains a very short pneumatic duct, much like a bowfin (*Amia calva*), though the highly compartmentalized nature is more reminiscent of a lungfish (Figure 1; Greenwood and Liem, 1984; Liem, 1989). Although the muscular sphincter controlling the pneumatic duct is not very impressive in juvenile arapaima, we found it to be substantial in size in a mature adult (Figure 3; Greenwood and Liem, 1984; Liem, 1989). Anatomical features of the slit-like aperture in the sphincter that *in vivo* could act as a valve to control the movement of air or limit water movement through the sphincter were seen in the adult specimen (Figures 3a–f).

### Renal System

Since the gills play a smaller role in homeostasis, air-breathing requires that the kidneys play a larger role in nitrogenous waste excretion, acid-base and ion regulation (Hochachka et al., 1978b; Brauner et al., 2004). Despite this, the gills are still the main site for urea and nitrogenous waste excretion (Gonzalez et al., 2010), and CO_2_. Hochachka et al. (1978b) reported the arapaima kidney is 3.5 times larger in relation to body mass than the closely related water-breathing aruana based on personal observation. We found the kidney to be 0.35% of total body volume, which is only slightly larger than the 0.26% of body volume seen in *O. mykiss*, a water-breathing fish (Table 1; Martinez et al., 2014). It is unclear if the aruana has unusually small kidneys in comparison to other teleosts, such as *O. mykiss*. Arapaima kidneys have no differentiation into head and trunk segments; nephrons are distributed throughout the parenchyma, along with hematopoietic cells, intrarenal chromaffin cells, melanomacrophage centers, lymphatic cells and corpuscles of Stannius (Figure 5b; Hochachka et al., 1978b). The nephron tubule has the same three segments as other fish, but the third segment is much longer and more developed (Hochachka et al., 1978b). The neck has tall cuboidal cells with large basal nuclei and the transition segment has an apical tubular system and a less-developed brush border (Hochachka et al., 1978b). The first segment and second segment have a complex basilar membrane network with oblong mitochondria, like in the water-breathing aruana (Hochachka et al., 1978b). The third segment is the longest in the arapaima and is made up of columnar cells with large basal nuclei and small oblong mitochondria, with an appearance very similar to the ionocytes prevalent in the epithelium of the gills (Hochachka et al., 1978b).

### Reproductive System

Fry production is the major restriction holding back arapaima aquaculture as reproduction is not as stable in captivity as in the wild and our understanding of arapaima reproduction is limited (Queiroz, 2000). Arapaima only have one developed ovary, the left, and they ovulate directly into the coelomic cavity as there is no oviduct (Godhino et al., 2005; Figures 5a,c,d). Eggs exit the coelomic cavity via a genital papilla that is approximately 6 mm in diameter in the caudal trunk (Godhino et al., 2005). There is considerable diversity in size at first maturity and nesting features for arapaima. This suggests that there may be multiple evolutionarily significant units or species (Godhino et al., 2005). The reported length at sexual maturity for females ranges from 137 to 207 cm depending on geographical site (Gurdak et al., 2019). At this length the females are approximately 5 years old, (Godhino et al., 2005), so our Specimen One was a mature adult. Arapaima are iteroparous but may not spawn every year. In years when they spawn, they often have multiple batches which reduce the risk of clutch failure (Queiroz, 2000). However as arapaima are partial spawners, they never have an empty ovary (Gurdak et al., 2019). With the start of the rising water season, reproductive activity begins and males and females pair off (Queiroz, 2000). The males build nests which are different in different regions. In the Lower Amazon, 90% of nests were found under woody vegetation (Gurdak et al., 2019). The males are responsible for caring for the young for approximately 4 months after breeding (Queiroz, 2000; Castello et al., 2011). Brood size per mating is currently unclear (Queiroz, 2000).

### Circulatory System

In order to facilitate these changes in gas bladder function, the vasculature pattern adapted in a variety of ways, most noticeably in regard to the gas bladder, the kidney, and the gills. The afferent blood supply to the gas bladder is via the dorsal aorta to the renal portal vein (Greenwood and Liem, 1984). The efferent blood vessels from the respiratory gas bladder are well developed, especially the left posterior cardinal veins which drain into the ductus Cuvier (Figure 6d; Hulbert and Moon, 1978; Greenwood and Liem, 1984; Graham, 1997). Pulmonary veins that return oxygenated blood directly to the sinus venosus are lacking.

All fish hearts are comprised of two compartments, the atrium and the ventricle (Figure 6; Icardo et al., 2005; Grimes and Kirby, 2009; Ishimatsu, 2012). Blood enters the heart via the sinus venosus, a thin walled compartment with minimal myocardium (Icardo, 2006). As in most air-breathing fish, there is mixing of deoxygenated systemic blood and oxygenated blood from the gas bladder as both the hepatic vein and the ductus Cuvier empty into the heart at the sinus venosus, eroding the efficiency of gas exchange (Farrell, 1978; Greenwood and Liem, 1984; Burggren and Johansen, 1986; Graham, 1997). Some highly specialized air-breathing fish have specific cardiac modifications to decrease this mixing, such as the partial interventricular septum seen in lungfish (Fishman et al., 1985; Icardo et al., 2005; Grimes and Kirby, 2009). Mixing is limited in the arapaima because most blood returning from the posterior systemic circulation comes from the renal portal system and goes through the respiratory gas bladder before emptying into the heart (Johansen et al., 1978; Greenwood and Liem, 1984). The oxygen partial pressure in the dorsal aorta of arapaima is 45–50 mmHg, which is an oxygen saturation of 80–90% (Johansen et al., 1978). Schaller and Dorn (1973) made note of a balloon valve at the entry point of the hepatic vein into the sinus venosus, which could limit mixing of saturated and unsaturated blood. We did not find evidence of this valve.

Arapaima have a type 1 heart, as outlined in Grimes and Kirby (2009), with a completely trabeculated ventricle and no coronary vasculature (Figure 6b). Like most fish with an entirely trabeculated ventricle, arapaima have a distinct muscular conus arteriosus which supports the conus valve (Figures 6a,b,d; Icardo, 2006). In some air-breathing fish, the conus is well developed (e.g., gars (Lepisosteriformes), bichers and lungfish), while others [e.g., snakehead (Channa argus) and climbing perch (Anabas testudineus)] have a regressed conus arteriosus (Ishimatsu and Itazawa, 1983; Grimes and Kirby, 2009). However, prior to Icardo (2006), the outflow tract of fish was poorly defined so it is possible that the conus arteriosus in the snakehead and climbing perch was simply identified using a different paradigm. The thick fibroelastic wall of the bulbus arteriosus allows it to stretch and dampen systolic blood pressure, functioning as a “windkessel” vessel as in other species (Figures 6a–d; Grimes and Kirby, 2009). The bulbus arteriosus has elaborate tissue folds filling the lumen (Figure 6b). Specialization of the bulbus arteriosus and ventral aorta have been seen in other air-breathing fish and can allow for more control of blood flow. Lungfish have spiral and ventrolateral folds inside the bulbus arteriosus that aid in directing blood flow either systemically or to the gills (Parsons, 1929; Fishman et al., 1985; Graham, 1997; Ishimatsu, 2012). The air-breathing snakehead, has smooth muscle ridges in the bulbus arteriosus and a bifurcated ventral aorta, one half of which directs blood to the gills and air-breathing organ and the other systemically (Ishimatsu et al., 1979; Ishimatsu and Itazawa, 1983; Grimes and Kirby, 2009). The climbing perch, African knifefish (*Gymnarchus niloticus*), swamp eel (*Monopterus cuchia*), and Indian catfish (*Heteropneustes fossilis*) are also all air-breathing fish with ridges in the bulbus arteriosus (Parsons, 1929; Munshi et al., 1986; Olson et al., 1994; Grimes and Kirby, 2009). At this point, it is unclear if the luminal folds in the bulbus arteriosus of the arapaima play a role in maximizing oxygen delivery efficacy, although studies of the oxygen and carbon dioxide partial pressures in the dorsal aorta showed no evidence of separation of blood flow based on oxygenation (Johansen et al., 1978; Randall and Farrell, 1978).

The heart of the arapaima is larger than that of the closely-related aruana, and is surrounded by a thick layer of lipid stores (Hochachka et al., 1978a). In the aruana, the whole heart makes up 0.025% of body weight, while in the arapaima the ventricle alone accounts for 0.1% of body weight (Hochachka et al., 1978a). We found the volume of the heart to be 0.21% of total volume (Table 1). The presence of lipid-rich connective tissue around the heart could act as a supplementary energy source that the majority of fish would be prohibited from using due to hypoxic conditions (Hochachka et al., 1978a).

The microvascularization of the gills also reflects changes associated with air-breathing. The branchial arteries have thick layers of smooth muscle, and each branch of the arterioles serves a larger area and some of the vessels are deeper within the tissue, reducing the amount of gas exchange (Hulbert and Moon, 1978). Together these changes indicate that there is a cardiac bypass system that allows the blood to skip the lamellae, controlling the amount of cardiac output devoted to the gills and decreasing oxygen loss to hypoxic water (Hulbert and Moon, 1978). Similar changes to the gill vascularization can be seen in the air-breathing snakehead and swamp eel (*Monopterus cuchia*) (Olson et al., 1986).

### Gastrointestinal System

Arapaima are an omnivorous species that mainly subsists on detritivorous and omnivorous species, such as catfishes and knifefishes, in the high-water season and macroinvertebrates in times of lower water (Watson et al., 2013). Grossly, the gastrointestinal tract can be divided in to four sections: the head gut is made up of the mouth and pharynx, the foregut is made up of the esophagus and stomach, the midgut is the longest section of the intestines, and the hindgut is the most terminal section including the rectum (Khojasteh, 2012).

The parasphenoid, vomer, premaxilla, maxilla, and dentary bones all bear teeth (Ridewood, 1905; Kershaw, 1976). The boney tongue, from which Osteoglossids derive their name, is made up of fine villiform lingual teeth from the medial hyobranchial bones to basibranchial toothplate (Figure 6; Ridewood, 1905; Stewart, 2013b; Watson et al., 2013). The boney tongue allows arapaima to crush the boney armor that protects catfish from most predators, enabling them to exploit an abundant food source (Watson et al., 2013).

The esophagus connects dorsally to the respiratory air bladder via a muscular sphincter. The stomach is where chemical digestion begins (Khojasteh, 2012). The intestine is 145% the total length of the fish, which is longer than would be expected for a carnivore (20% total length) and shorter than seen in herbivores (up to 2000% total length) (Khojasteh, 2012; Watson et al., 2013). In arapaima, the intestines loop back and forth within the coelom, in contrast to the carnivorous *O. mykiss*, where the intestine is a short straight tube (Khojasteh, 2012). Arapaima have two pyloric ceca, like most osteoglossid fish (Khojasteh, 2012; Watson et al., 2013). The gut of the osteoglossomorpha is distinct from that of other fishes in that the intestine passes posteriorly to the left of the esophagus and stomach (Nelson, 1972) rather than to the right (Figures 7G,H).

The volume of the liver of the arapaima was 0.57% of total body volume, compared to 0.43% in *O. mykiss* (Table 1). It is difficult to draw conclusions based on liver size in fish, as there is substantial interspecies variability as well as intraspecies variability based on sex, age, season and body condition (Datta-Mushi and Dutta, 1996).

### Nervous System

There are relatively few studies looking at teleost brains compared to other clades and using MR is uncommon (Ullmann et al., 2010). As a result of their taxonomic breadth and the variety of ecological niches they fill, teleost brains are hugely morphologically variable to reflect the unique needs of each species (Huber et al., 1997; Salva et al., 2014). Most teleosts follow the standard configuration, where the spinal cord attaches to the brainstem, mesencephalon and diencephalon, with the mesencephalon made up primarily of the cerebellum and optic lobes (Figure 8). The telencephalon is made up of the cerebral hemispheres with the olfactory bulbs attached rostrally (Kotrschal et al., 1998). The braincase is often much larger than the brain, so brain size is not restricted (Kotrschal et al., 1998). The excess space is often filled with lymphatic fatty tissue (Kotrschal et al., 1998). In terms of brain-to-body weight, arapaima and other osteoglossids have a high degree of encephalization compared to many fish, comparable to that of a tuna or a jackfish (Bauchot et al., 1994). We found the volume of the brain to be 0.01% of total body volume (Table 1). Bauchot et al. (1994) reported that osteoglossids have very small olfactory bulbs compared to other freshwater fish making them microsmatic, but we found the olfactory bulbs to be twice the reported values for *O. mykiss* and 2.5 times the value reported for arapaima (Bauchot et al., 1994; Martinez et al., 2014). This is interesting because Bauchot et al. (1994) used juvenile arapaima in their study, so this difference may reflect an increase in the importance of olfaction as arapaima mature to become more migratory predators. In addition as seen in Bauchot et al. (1994), the size of the brain does not increase proportionately with the muscle volume of the fish as they age, resulting in younger fish brains constituting a larger percentage of total body volume. The cerebral hemispheres are large in the arapaima and may have some olfactory function as well as in coordinating motor centers (Bauchot et al., 1994). The telencephalon, is large, making up 28.3% (Table 3) of brain volume in our specimen, compared to other teleost species that have a mean telencephalon volume of 15.5% total brain volume. It is unclear why arapaima have such well-developed telencephalons, but it may be related to their migratory nature, high degree of parental care and piscivorous hunting. From our imaging data, the demarcation between optic tectum and the tectum mesencephalicum was unclear. In total they measured 27.89% of total volume with is similar to values (28.78%) for arapaima (Bauchot et al., 1994). The valvula cerebelli, a component of the tectum mesencephalicum along with the optic tectum, has gustatory and motor activity control functions (Bauchot et al., 1994). Like many basal fish, the arapaima has a relatively small cerebellum comprising 16.37% of brain volume in our specimen (Table 3). This relates to their low activity lifestyles in shallow water, combined with bursts of movement for hunting (Bauchot et al., 1994; Kotrschal et al., 1998).

### Endocrine System

In our specimen, the pituitary was 1.77% of the volume of the brain (Table 3). The pituitary of arapaima is highly vascular, with the neurohypophysis and pars distalis being very well connected by what could be considered a hypophyseal-portal system (Borella et al., 2009). The neurohypophysis is dorsal to the pars distalis (Borella et al., 2009). The pars distalis contains ACTH, gonadotropin, prolactin, and growth hormone secreting cells, while the pars intermedia contains cells that can produce melanotropin, adrenocorticotropin and somatolactin (Borella et al., 2009).

### Musculoskeletal System and Integument

When preparing an arapaima for human consumption, the scales, skin and fins are removed (Queiroz, 2000). Then a longitudinal cut is made along the spine (Queiroz, 2000). The ribs and remaining fin rays can then be loosened from the flesh leaving a triangular boneless piece of meat called the “manta” (Queiroz, 2000). The middle of this slab, the “ventrecha,” is a highly prized delicacy (Queiroz, 2000). As total muscle makes up near to 82% of the volume of the arapaima, fishing for arapaima is a lucrative pursuit (Table 1).

As with most teleosts, arapaima swim via undulation, which is achieved through conical formation of the myomeres (Müller and Van Leeuwen, 2006). Myomeres contract in waves causing the body to bend (Wardle et al., 1995). This form of swimming allows the entire body to be used to create thrust (Müller and Van Leeuwen, 2006).

The muscle tissue of the arapaima is uniquely modified to have increased aerobic and decreased anaerobic capabilities compared to water-breathing fish (Hochachka and Guppy, 1978). Like the closely-related jeju (*Hopletrythrinus unitaeniatus*), arapaima have mostly white muscle fibers but, unlike the jeju, arapaima have retained some red muscle fibers in small dorsal and lateral superficial bands which have a higher metabolism and require more oxygen, which the arapaima provides through air-breathing (Hochachka and Guppy, 1978). Red muscle is used to power continuous swimming, while white muscle is for burst swimming (Müller and Van Leeuwen, 2006). The white muscle in the arapaima has a low metabolism, which allows the arapaima to maintain a low overall metabolism (Hochachka and Guppy, 1978). This drive to conserve oxygen between breaths is evidenced by their sluggish movements between breaths, interspersed with bursts of predatory action (Almeida-Val and Hochachka, 1995). Their decreased reliance on anaerobic metabolism creates less lactic acid build up, allowing them to make each breath last longer underwater in a manner similar to diving mammals (Almeida-Val and Hochachka, 1995).

Arapaima have elongated skulls that retain many of the basal characteristics of actinopterygians but are still highly modified for their environment (Hilton et al., 2007; Figures 9A–C). The head is very flat dorsally with the eyes positioned in line with the top of the top of the head, which aids in breathing while minimally emerging from the surface of the water. The nasal bones are large and, like the frontal, parietal, pterotic and preopercular bones, have shallow depressions which surround the pores of the acousticolateralis system (Kershaw, 1976). These depressions are most evident in the preopercular bone, where there are three distinct elliptical hollows (Kershaw, 1976). The dermethmoid bone is rhomboidal, similar to other osteoglossids, but larger (Kershaw, 1976). The occipito-vertebral region of the arapaima is unique to the genus and can be used to identify specimens from the fossil record (Hilton et al., 2007). The parapophyses of the first vertebral centrum are enlarged and fused to the centrum, where they extend to the parasphenoid (Hilton et al., 2007). The bones of the cranium, as well as the pectoral girdle, play a role in creating the bucco-pharyngeal suction that allows arapaima to catch prey that are often faster and more agile (Kershaw, 1976).

Arapaima also have a flexible dermal armor of elasmoid scales made from overlapping layers of type 1 collagen and a highly mineralized hydroxyapatite outer layer (Yang et al., 2014). As in the heavily scaled gar, the flexural stiffness of the scales in the arapaima likely play an important role in the mechanics of undulatory swimming (Long and Nipper, 1996). Some of these scales are modified with an opening in the posterior third to accommodate the lateral line system (Stewart, 2013b). Scales near the caudal portion of the cranium at the nape insert into the parietal bone (Figure 9; Stewart, 2013b).

## Summary

There are some acknowledged limitations of the current study. As adult arapaima are rare, only one specimen was available for CT and MR analysis. Although the primary specimen displayed some pathological changes such as pericardial, gas bladder and abdominal effusion and fibrosing cardiomyopathy, these changes did not drastically alter the morphometric analysis. Ideally, future quantitative research will be done on arapaima of varying sizes, sex, age, and species in order to gain more representative data.

Morphometric analysis from CT and MR data is subject to error associated with post-mortem processes and segmentation. Post-mortem changes were minimized by imaging within 24 h of death and by imaging fresh tissues (Berquist et al., 2012). Errors in the manual segmentation of imaging data into structures was minimized through reference to necropsy photographs and various arapaima specific literature.

As in many species, the major threats facing wild arapaima today are overfishing, climate change, and environmental degradation (Castello and Stewart, 2010; Freitas et al., 2013). Wild arapaima are especially prone to overfishing due to both their desirability as prey and their life history. Arapaima are easy to hunt with harpoons and gillnets as they periodically rise to the surface, and they are viewed as trophy species due to their extreme size and the fact they produce high quality meat (Castello and Stewart, 2010; Castello et al., 2011). In some areas of the Amazon basin, arapaima make up 47% of income from fishing and 70% of arapaima caught are below the minimum allowed size (Queiroz, 2000). Their populations are slow to recover from overfishing due to their size, high parental input to young, small clutch sizes, and high age at reproductive maturity (Castello et al., 2011). Despite some protection under ICUN and CITES, there is little enforcement of size and season regulations, or moratoriums (Castello and Stewart, 2010). Fortunately arapaima are increasingly be raised in aquaculture as both commercially and also as part of conservation (Schaefer et al., 2012).

Climate change is an increasing concern in the Amazon basin, where there is the highest fish diversity in the world, and it is expected to cause a 7–12% loss of fish species over the next 50 years (Freitas et al., 2013). The arapaima life cycle is heavily influenced by the annual hydrologic cycle of flooding and receding waters when they migrate between lakes, rivers and flooded forests (Queiroz, 2000; Castello, 2008). During times of low water, arapaima can become stranded in smaller lakes with deteriorating water quality where they are easy prey for fishermen (Queiroz, 2000). During the extreme drought brought about by a warming Atlantic in 2005, the population of arapaima’s closest relation in the Amazon, the aruana (*Osteoglossum bicirrhosum*), was decreased by over 50% with only a partial recovery by 2007 (Freitas et al., 2013). Arapaima are likely to be more heavily impacted by climate change as they are carnivores rather than planktivores or detritovores, but their annual migration between the lakes and rivers may be protective (Freitas et al., 2013). Although the impact of climate change is dire, direct human impact from activities such as agriculture, mining, urbanization, pollution, and hydroelectric dams are expected to have larger consequences in the Amazonian wetland ecosystem in the coming years (Junk, 2013).

## Data Availability Statement

The raw data supporting the conclusions of this article will be made available by the authors, without undue reservation, to any qualified researcher.

## Ethics Statement

All specimens were owned by SeaWorld and provided by Dr. St. Leger who was the director of research at SeaWorld San Diego.

## Author Contributions

JS and MS conceived the project. MS acquired the MRI data. MS, CM, WH, DS, DD, and HB contributed to image data segmentations and designed and produced the figures. CM, MS, and JS, drafted the manuscript. All authors contributed to the final manuscript.

## Conflict of Interest

MS and DD are partners and work at the same institutions. JS worked at SeaWorld when project was started. The remaining authors declare that the research was conducted in the absence of any commercial or financial relationships that could be construed as a potential conflict of interest.
